# Supplementary material for: BMP3 Deficiency Accelerates Cartilage-to-Bone Transition in Ectopic Bone
Source: Biomedicines. 2025 Oct 15;13(10):2508. doi: 10.3390/biomedicines13102508 (PMC12561720; doi:10.3390/biomedicines13102508)
Supplement: Supplementary file 1 [file biomedicines-13-02508-s001.zip › biomedicines-3908084-supplementary.pdf]

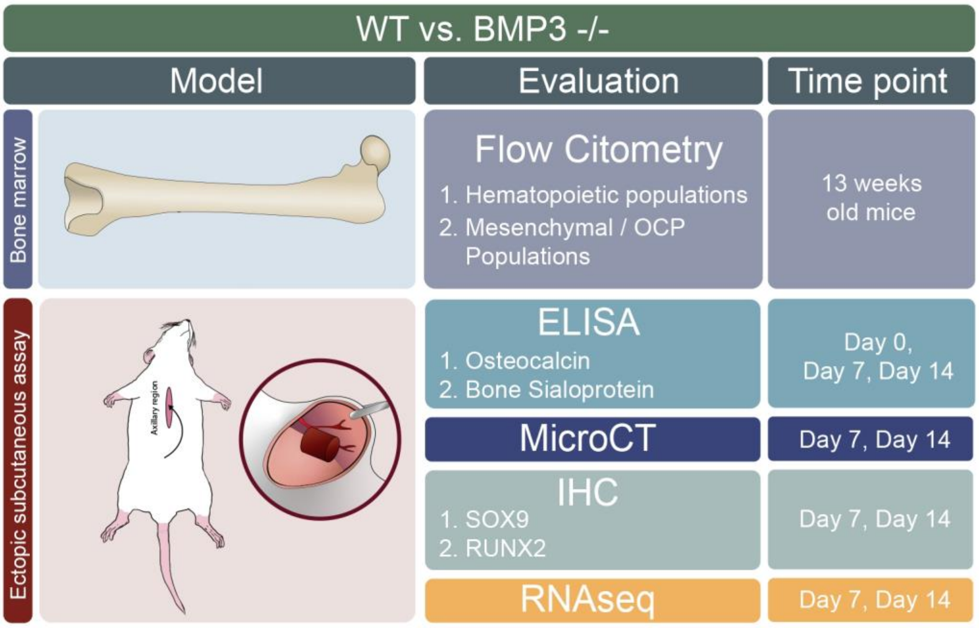

Figure S1. Experimental design. Methodology used for obtaining results on the influence of BMP3 on ectopic bone formation. Fluorescece-activated cell sorting (FACS) was used to analys osteoprogenitor cells in bone marrow of long bones (A). Subcutaneous ectopic bone model was used to evaluate bone formation by micro-CT, immunohistochemistry and RNAseq methods (B).

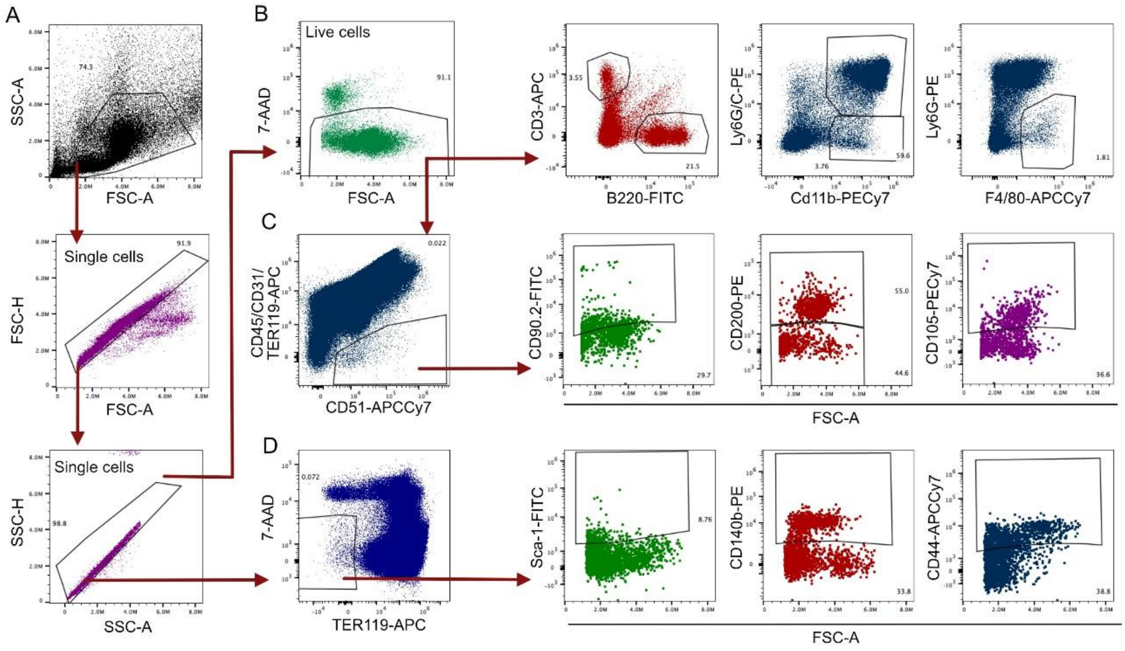

Figure S2. Schematics of flow cytometry analysis. (A**)** All analyses were performed exclusively on single, live cells (7-AAD$^-$). Hematopoietic lineages (B) were delineated according to the expression of B220, CD3, Cd11b, Ly6G/Ly6C and F4/80. Mesenchymal lineage cells were labeled with 2 antibody panels. Panel 1 included antibodies to CD90.2, CD200, and CD105 markers, whose expression was assessed on single, live, non-hematopoietic (CD45$^-$TER119$^-$ CD31$^-$) CD51$^+$ cells, and panel 2 antibodies to Sca-1, CD140b, and CD44, that were assessed among live, non-hematopoietic (CD45$^-$TER119$^-$CD31$^-$) cells. Dead cells were excluded by binding of 7-AAD. Proportions of positive cells were delineated according to the signal of non-stained cells or appropriate fluorescence minus one (FMO) controls.

Table S1. Top 50 differentially expressed genes in Bmp3$^{-/-}$ samples in comparison with WT at day 7.

Rank	Gene abbreviation	Gene name	Mean counts	Log_2 Fold change
1	Bmp3	Bone morphogenetic protein 3	223	-5.66
2	Adgrf4	Adhesion G protein-coupled receptor F4	131	3.66
3	Ngp	Neutrophilic granule protein	1513	4.22
4	Olfm2	Olfactomedin 2	69	6.34
5	Bglap	Bone gamma carboxyglutamate protein	229	5.96
6	Slc26a7	Solute carrier family 26, member 7	1452	3.72
7	Msx2	Msh homeobox 2	55	3.11
8	Oscar	Osteoclast associated receptor	109	5.3
9	Slc9b2	Solute carrier family 9, subfamily B	396	3.54
10	Ppp1r27	Protein phosphatase 1, regulatory subunit 27	21	-4.57
11	Bglap2	Bone gamma-carboxyglutamate protein 2	63	4.72
12	Mmrn1	Multimerin 1	220	-2.63
13	Dcstamp	Dendrocyte expressed seven transmembrane protein	98	4.37
14	Tspan15	Tetraspanin 15	57	-3.38
15	Acp5	Acid phosphatase 5, tartrate resistant	1250	4.48
16	Tuba8	Tubulin, alpha 8	72	-2.9
17	Pard6b	PAR6 family cell polarity regulator beta	25	0.37
18	Smim7	Small integral membrane protein 7	382	-0.36
19	Liph	Lipase H	45	-0.44

20	Swi5	SWI5 recombination repair homolog (yeast)	1396	-0.15
21	Cnrip1	cannabinoid receptor interacting protein 1	211	0.01
22	Csrnp2	cysteine and serine rich nuclear protein 2	93	0.4
23	Gm11507	Predicted gene 11507	3	0
24	Lppr4	Phospholipid phosphatase related 4	160	-0.2
25	Gskip	GSK3B interacting protein	158	0.02
26	Cnpy1	Canopy FGF signaling regulator 1	53	0.28
27	2010007H06Rik	RIKEN cDNA 2010007H06 gene	69	0.52
28	Palb2	Partner and localizer of BRCA2	136	0.24
29	Phf11a	PHD finger protein 11A	39	-0.56
30	Gemin7	Gem nuclear organelle associated protein 7	53	-0.08
31	Slc38a6	Solute carrier family 38, member 6	531	0.48
32	Dnd1	DND microrna-mediated repression inhibitor 1	19	0.49
33	Gpr152	G protein-coupled receptor 152	16	-0.26
34	Fzd1	Frizzled class receptor 1	433	0.12
35	Tlr7	Toll-like receptor 7	465	-0.32
36	Tram1l1	Translocation associated membrane protein 1-like 1	61	-0.19
37	5031434C07Rik	RIKEN cdna 5031434C07 gene	21	0.34
38	Zfp507	Zinc finger protein 507	439	0.26
39	Ffar1	Free fatty acid receptor 1	25	0.38
40	Fam160a2	FHF complex subunit HOOK interacting protein 1B	476	-0.08

41	Fam46c	Terminal nucleotidyltransferase 5C	652	0.14
42	Lincpint	Long non-protein coding RNA, Trp53 induced transcript	152	-0.34
43	Ascc1	Activating signal cointegrator 1 complex subunit 1	92	0.23
44	Klk1b11	Kallikrein 1-related peptidase b11	9	-0.57
45	2510039O18Rik	RIKEN cdna 2510039O18 gene	140	0.45
46	Bod1	Biorientation of chromosomes in cell division 1	162	-0.56
47	Foxe3	Forkhead box E3	3	0
48	Znrf4	Zinc and ring finger 4	3	0
49	Klk14	Kallikrein related-peptidase 14	16	0.04
50	Obox3-ps8	Oocyte specific homeobox 3, pseudogene 8	8	0

Table S2. Top 50 differentially expressed genes in Bmp3$^{-/-}$ samples in comparison with WT at day 14.

Rank	Gene abbreviation	Gene name	Mean counts	Log_2 Fold change
1	Ngp	Neutrophilic granule protein	1513	-8.54
2	Ppbp	Pro-platelet basic protein	674	-6.53
3	Slc25a37	Solute carrier family 25, member 37	881	-4.02
4	Slc4a1	Solute carrier family 4 (anion exchanger), member 1	1106	-5.28
5	Fam46c	Terminal nucleotidyltransferase 5C	652	-4.05
6	Lcn2	Lipocalin 2	359epb	-5.2

7	Prg2	Proteoglycan 2, bone marrow	233	-5.42
8	Mpo	Myeloperoxidase	482	-4.93
9	Gypa	Glycophorin A	254	-4.28
10	Hbb-bt	Hemoglobin, beta adult t chain	956	-5.66
11	S100a9	S100 calcium binding protein A9 (calgranulin B)	1294	-7.24
12	Retnlg	Resistin like gamma	203	-5.28
13	Camp	Cathelicidin antimicrobial peptide	153	-4.73
14	Hbb-bs	Hemoglobin, beta adult s chain	1804	-4.66
15	Elane	Elastase, neutrophil expressed	146	-4.97
16	S100a8	S100 calcium binding protein A8 (calgranulin A)	1566	-6.76
17	Igkc	Immunoglobulin kappa constant	296	-4.95
18	Epb4.1	Erythrocyte membrane protein band 4.1	873	-2.89
19	Hmgb3	High mobility group box 3	386	-3.6
20	Alas2	Aminolevulinic acid synthase 2, erythroid	299	-4.36
21	Hemgn	Hemogen	206	-4.26
22	Tuba4a	Tubulin, alpha 4A	550	-3.7
23	Ermap	Erythroblast membrane-associated protein	245	-3.15
24	Lgals1	Lectin, galactose binding, soluble 1	2344	3.31
25	I830127L07Rik	Lymphocyte antigen 6 family member A2	67	-4.4
26	Tmcc2	Transmembrane and coiled-coil domains 2	312	-3.11
27	Cd177	CD177 antigen	223	-3.79
28	Ltf	Lactotransferrin	943	-5.44
29	Tal1	T cell acute lymphocytic leukemia 1	163	-3.14

30	Spta1	Spectrin alpha, erythrocytic 1	575	-2.7
31	Ctsg	Cathepsin G	84	-3.71
32	Mmp8	Matrix metallopeptidase 8	254	-3.22
33	Trim10	Tripartite motif-containing 10	146	-3.1
34	Sptb	Spectrin beta, erythrocytic	583	-3.19
35	Abcb10	ATP-binding cassette, sub-family B member 10	273	-2.56
36	Hmbs	Hydroxymethylbilane synthase	212	-2.87
37	Islr	Immunoglobulin superfamily containing leucine-rich repeat	1468	3.1
38	Snca	Synuclein, alpha	198	-3.55
39	Tfrc	Transferrin receptor	1878	-2.75
40	E2f2	E2F transcription factor 2	229	-3.67
41	Rsad2	Radical S-adenosyl methionine domain containing 2	421	-3.41
42	Col6a3	Collagen, type VI, alpha 3	17238	2.32
43	Cthrc1	Collagen triple helix repeat containing 1	2829	3.8
44	Ctss	Cathepsin S	1769	2.32
45	Chil3	Chitinase-like 3	455	-3.65
46	Lmnb1	Lamin B1	705	-2.34
47	Fn1	Fibronectin 1	23636	2.65
48	Apol11b	Apolipoprotein L 11b	144	-3.13
49	Acan	Aggrecan	2586	3.32
50	Pglyrp1	Peptidoglycan recognition protein 1	80	-4.04

Table S3. Gene ontology and pathway analysis of ectopic bone on day 7 post-implantation in Bmp3$^{-/-}$ and WT mice.

Rank	Gene set name	Gene set category	Adjusted p-value	Expression pattern
1	negative regulation of lymphangiogenesis	GO:Biological Process	0.198	Higher in $Bmp3^{-/-}$
2	regulation of lymphangiogenesis	GO:Biological Process	0.346	Higher in $Bmp3^{-/-}$
3	Cytokine-cytokine receptor interaction	KEGG Pathway	0.536	Higher in WT
4	lymphangiogenesis	GO:Biological Process	0.939	Higher in $Bmp3^{-/-}$
5	RNA biosynthetic process	GO:Biological Process	1	Higher in WT
6	developmental process	GO:Biological Process	1	Higher in WT
7	multicellular organismal process	GO:Biological Process	1	Higher in WT
8	cell differentiation	GO:Biological Process	1	Higher in WT
9	signaling	GO:Biological Process	1	Higher in WT
10	regulation of metabolic process	GO:Biological Process	1	Higher in WT
11	regulation of nucleobase-containing compound metabolic process	GO:Biological Process	1	Higher in WT
12	RNA metabolic process	GO:Biological Process	1	Higher in WT
13	positive regulation of macromolecule metabolic process	GO:Biological Process	1	Higher in WT
14	positive regulation of macromolecule biosynthetic process	GO:Biological Process	1	Higher in WT
15	regulation of gene expression	GO:Biological Process	1	Higher in WT
16	nucleobase-containing compound biosynthetic process	GO:Biological Process	1	Higher in WT
17	gene expression	GO:Biological Process	1	Higher in WT
18	cellular process	GO:Biological Process	1	Higher in WT

19	positive regulation of metabolic process	GO:Biological Process	1	Higher in WT
20	positive regulation of biosynthetic process	GO:Biological Process	1	Higher in WT
21	regulation of biosynthetic process	GO:Biological Process	1	Higher in WT
22	tissue development	GO:Biological Process	1	Higher in WT
23	macromolecule biosynthetic process	GO:Biological Process	1	Higher in WT
24	biosynthetic process	GO:Biological Process	1	Higher in WT
25	metabolic process	GO:Biological Process	1	Higher in WT
26	regulation of macromolecule biosynthetic process	GO:Biological Process	1	Higher in WT
27	macromolecule metabolic process	GO:Biological Process	1	Higher in WT
28	primary metabolic process	GO:Biological Process	1	Higher in WT
29	positive regulation of DNA-templated transcription	GO:Biological Process	1	Higher in WT
30	regulation of RNA biosynthetic process	GO:Biological Process	1	Higher in WT

Table S4. Gene ontology and pathway analysis of ectopic bone on day 14 post-implantation in $Bmp3^{-/-}$ and WT mice.

Rank	Gene set name	Gene set category	Adjusted p-value	Expression pattern
1	immune system process	GO:Biological Process	0	Higher in WT
2	defense response	GO:Biological Process	0	Higher in WT
3	regulation of immune system process	GO:Biological Process	0	Higher in WT
4	defense response to other organism	GO:Biological Process	0	Higher in WT

5	response to stress	GO:Biological Process	0	Higher in WT
6	defense response to symbiont	GO:Biological Process	0	Higher in WT
7	response to other organism	GO:Biological Process	0	Higher in WT
8	response to external biotic stimulus	GO:Biological Process	0	Higher in WT
9	Metal sequestration by antimicrobial proteins	Reactome Pathway	0	Higher in WT
10	myeloid cell differentiation	GO:Biological Process	0	Higher in WT
11	regulation of multicellular organismal process	GO:Biological Process	0	Higher in WT
12	response to biotic stimulus	GO:Biological Process	0	Higher in WT
13	biological process involved in interspecies interaction between organisms	GO:Biological Process	0	Higher in WT
14	hemopoiesis	GO:Biological Process	0	Higher in WT
15	Neutrophil degranulation	Reactome Pathway	0	Higher in WT
16	immune response	GO:Biological Process	0	Higher in WT
17	response to external stimulus	GO:Biological Process	0	Higher in WT
18	positive regulation of immune system process	GO:Biological Process	0	Higher in WT
19	response to lipopolysaccharide	GO:Biological Process	0	Higher in WT
20	erythrocyte homeostasis	GO:Biological Process	0	Higher in WT
21	response to molecule of bacterial origin	GO:Biological Process	0	Higher in WT
22	innate immune response	GO:Biological Process	0	Higher in WT
23	Antimicrobial peptides	Reactome Pathway	0	Higher in WT

24	leukocyte migration involved in inflammatory response	GO:Biological Process	0	Higher in WT
25	response to bacterium	GO:Biological Process	0	Higher in WT
26	cellular oxidant detoxification	GO:Biological Process	0	Higher in WT
27	erythrocyte development	GO:Biological Process	0	Higher in WT
28	porphyrin-containing compound biosynthetic process	GO:Biological Process	0	Higher in WT
29	tetrapyrrole biosynthetic process	GO:Biological Process	0	Higher in WT
30	myeloid cell homeostasis	GO:Biological Process	0	Higher in WT
